# Effects of butter from mountain-pasture grazing cows on risk markers of the metabolic syndrome compared with conventional Danish butter: a randomized controlled study

**DOI:** 10.1186/1476-511X-12-99

**Published:** 2013-07-10

**Authors:** Louise B Werner, Lars I Hellgren, Marianne Raff, Søren K Jensen, Rikke A Petersen, Tue Drachmann, Tine Tholstrup

**Affiliations:** 1 Department of Nutrition, Exercise and Sports, Faculty of Sciences, University of Copenhagen, Frederiksberg 1958, Denmark; 2 Center for Biological Sequence Analysis, DTU Systems Biology, Technical University of Denmark, 2800 Lyngby, Denmark; 3 Department of Animal Health and Bioscience, Faculty of Agricultural Sciences, Aarhus University, 8830 Tjele, Denmark

**Keywords:** Dairy-fat, Low-input system, Phytanic acid, LDL cholesterol, Oral glucose tolerance test

## Abstract

**Background:**

There is considerable interest in dairy products from low-input systems, such as mountain-pasture grazing cows, because these products are believed to be healthier than products from high-input conventional systems. This may be due to a higher content of bioactive components, such as phytanic acid, a PPAR-agonist derived from chlorophyll. However, the effects of such products on human health have been poorly investigated.

**Objective:**

To compare the effect of milk-fat from mountain-pasture grazing cows (G) and conventionally fed cows (C) on risk markers of the metabolic syndrome.

**Design:**

In a double-blind, randomized, 12-week, parallel intervention study, 38 healthy subjects replaced part of their habitual dietary fat intake with 39 g fat from test butter made from milk from mountain-pasture grazing cows or from cows fed conventional winter fodder. Glucose-tolerance and circulating risk markers were analysed before and after the intervention.

**Results:**

No differences in blood lipids, lipoproteins, hsCRP, insulin, glucose or glucose-tolerance were observed. Interestingly, strong correlations between phytanic acid at baseline and total (P<0.0001) and LDL cholesterol (P=0.0001) were observed.

**Conclusions:**

Lack of effects on blood lipids and inflammation indicates that dairy products from mountain-pasture grazing cows are not healthier than products from high-input conventional systems. Considering the strong correlation between LDL cholesterol and phytanic acid at baseline, it may be suggested that phytanic acid increases total and LDL cholesterol.

**Trial registration:**

ClinicalTrials.gov, NCT01343589

## Background

Increased consumer awareness of the link between diet and health has led to research focused on altering the fatty acid (FA) composition of cows’ milk to achieve a FA profile consistent with public health recommendations. However, as modification of the FA content of milk fat in dairy cows is affected significantly by the extensive metabolism of lipids that occurs in the rumen, it is important to understand the interrelationship between dietary supply of lipids, rumen fermentation, and mammary synthesis of milk fat. Important targets include reducing the amount of long-chain saturated fatty acids (SFA’s) such as C12:0-C16:0, enhancing oleic acid to reduce cardiovascular risk, and generally increasing concentrations of mono- and poly-unsaturated fatty acid (MUFA and PUFA) [1-10].

It is well known that the FA composition of milk fat is markedly influenced by the feed given to the dairy cows [11-14]. The addition of forage, especially fresh grass, to fodder has been found to enhance the proportion of unsaturated FAs in milk fat [15-17], and to elevate the concentration of the *c-*9, *t-*11 isomer of conjugated linoleic acid (CLA) [17,18]. Unlike the *c-*10, *t-*12 isomer of CLA, which in animal models has proved to be a biologically active FA with beneficial effects on diseases such as cancer, diabetes, and obesity [19-21], the *c*-9, *t-*11 CLA appears to be neutral [22-27]. However, the markedly higher intake of chlorophyll-containing green fodder in grazing cows also results in a high phytanic acid content in milk from these cows [28,29]. Phytanic acid may be an interesting, though so far overlooked, bioactive FA with potential positive effects on metabolic function. Phytanic acid (3,7,11,15-tetramethyl hexadecanoic acid) is a multi-branched FA with reported retinoid X receptor (RXR) and peroxisome proliferator activated receptor-*α* (PPAR-*α*) agonist activity [30-33]. It has been suggested that, due to its reported RXR and PPAR-α agonist activity and its ability to induce glucose uptake in hepatocytes, phytanic acid may prevent metabolic dysfunctions related to the development of metabolic syndrome [34].

The purpose of this study was to investigate the effects on risk markers of cardiovascular diseases (CVD) and type-2 diabetes in healthy humans of a diet containing milk delivered from mountain-pasture grazing cows from Norway (G) with that of a diet containing milk fat of typical Danish composition (C). Although, modification of the dairy cows’ diet has been extensively studied, only very few human dietary intervention studies have examined the effect of these modified dairy products on CVD risk. In addition, the actual effects on human health of milk from pasture-grazing cows and conventional Danish milk have been poorly investigated. Since no data exists on the effects of intake of phytanic acid in a normal diet, we have also paid particular attention on the potential effects of this FA.

## Subjects and methods

### Study design

We conducted a 12-week, controlled, double-blind, randomized, parallel intervention study. Participants were provided with the test diets that substituted part of their daily diet and replaced approximately 15 percent of energy (E%) of their daily fat intake. Participants were stratified according to baseline blood glucose level, gender, and waist circumference, into two random treatment groups receiving either the G diet or the C diet.

### Subjects

Subjects ranged in the age from 50 to 70 years old, and were recruited for the study through ads placed in local newspapers. The baseline characteristics of the 38 subjects who completed the study are given in Table 1. Exclusion criteria were: BMI > 35 kg/m^2^, current or previous chronic disease, regular use of medication, or drug and alcohol abuse. All participants were apparently healthy as indicated by a medical and lifestyle questionnaire. They all agreed to refrain from taking any dietary supplements, from donating blood two months before and during the study, and from taking any medication that might interfere with study measurements. All subjects were instructed to maintain the same level of physical activity throughout the study. Subjects completed a three-day dietary record in order to provide information about dietary intake during the intervention. This was done before the intervention and after six weeks of intervention. We found it relevant to evaluate the diet after six weeks to gain information about dietary intake during the intervention. Two weekdays and one Saturday/Sunday were included in the dietary record to take into account any differences in nutrient intake during weekdays and the weekend. After they had delivered their first dietary record, participants were given feedback and advice on how they could decrease fat intake during the intervention period. This was achieved by guidance from our dietician. Collection of the test food as well as follow-up and weighing every second week took place at Department of Nutrition, Exercise and Sports. The subjects were also weighed before and after the intervention. In the event of weight gain, the participants were weighed each week and received advice from our dietician on how to maintain a stable weight. After four and eight weeks of intervention, the participants were interviewed about their well-being, physical activity level and diet to make sure they were adhering to the test diet and maintained the same weight and physical activity level. In addition, participants were supplied with recipes to provide inspiration on how the test diet could be consumed. To keep track of compliance with, and potential deviations from, the study guidelines, subjects were asked to keep a log book in which they recorded on a daily basis any illness, unusual activities, and failure to consume the test diet.

**Table 1:** **Baseline characteristics for the 38 healthy subjects participating in the 12 week intervention**^**1**^

	**Grazing ( *n *=20)**	**Conventional ( *n *=18)**
Women, (*n, %)*	13, 65%	10, 55.6%
Age, (*y)*	61.9 ± 4.9 (52–69)	60.7 ± 5.9 (50–69)
Height, (*cm)*	170.5 ± 8.4 (158–187)	173.2 ± 7.5 (159.8-188)
Weight, (*kg)*	73.6 ± 8.4 (56.3-93)	79.9 ± 15.5 (61.35-122.6)
BMI, (*kg/m2)*	25.4 ± 2.7 (20.81- 30.55)	26.5 ± 3.6 (21.22-34.69)
Total cholesterol, *(mmol/L)*	5.8 ± 1.15 (3.95-7.53)	5.5 ± 0.86 (3.98-7)
LDL cholesterol, *(mmol/L)*	3.3 ± 0.83 (1.46-4.53)	3.3 ± 0.59 (2.45-4.21)
HDL cholesterol, *(mmol/L)*	1.5 ± 0.29 (1.02-2.06)	1.3 ± 0.31 (0.75-1.73)
Triacylglycerol, (*mmol/L*)	1.2 ± 0.46 (0.76-2.62)	1.3 ±0.59 (0.73-2.74)
hsCRP, (*mg/L)*	1.2 ± 1.46 (0.05-5.42)	1.0 ± 0.93 (0.05-3.11)
Insulin, *(pmol/L)*	30.6 ± 20.05 (7.2-90.75)	41.4 ± 35.04 (7.2-127)
C-peptide, *(pmol/L)*	566.2 ± 224.03 (349.5-1065.5)	611.8 ± 287.82 (298–1299.5)
Glucose, *(mmol/L)*	5.6 ± 0.51 (5.02-7.14)	5.7 ± 0.47 (5.14-6.57)
Phytanic acid, (*μM)*	3.0 ± 0.69 (1.98-4.76)	3.0 ± 0.61 (2.3-4.28)

^1^ Values are mean ± SD; range in parentheses. *hsCPR* high sensitive C-reactive protein, *Grazing* milk from grazing cows, *Conventional* milk from conventionally fed cows.

Dietary intake was estimated using Dankost 3000 dietary assessment software (Dankost, Copenhagen, Denmark). Mean habitual energy intakes before the intervention were 8.6 MJ/d (range: 5.6-15.8 MJ/d); 33.5% (24.2-48.2%) of energy was from fat; 16.3% (11.6-22.8%) was from protein; and 45.7% (30–58.3%) was from carbohydrates. Carbohydrate intake in the G group (43.86 ± 5.47 E%) was reported to be lower than in the C group (47.84 ± 5.27 E%) (p=0.029) when expressed as E%, but not when expressed as g/d. There were no other differences in habitual dietary intake between the two groups. The protocol and aims of the study were fully explained (orally and in writing) to the participants, who subsequently gave written informed consent. The Scientific Ethics Committee for Copenhagen and Frederiksberg approved the research protocol (H-B-2009-052).

### Production of test milk

Milk fat for the human study was derived from milk from pasture-grazing cows (Norwegian red cattle breed) or conventionally fed Danish cows (Holstein-Friesian breed). The two types of milk produced differed in FA composition. The markedly higher intake of chlorophyll-containing green fodder of the grazing cows resulted in a high phytanic acid content in milk compared with the conventional Danish milk. The milk from grazing cows was produced by 104 cows from seven farms. It was collected in August late in the grazing-season, as it was assumed that the effects of grazing would be strongest when the cows had been grazing all summer. The conventional Danish milk was provided by Arla Food amba (Viby J, Denmark) and originated from 47 farms. It was collected in October when the cows were kept indoors and fed conventional winter fodder. Cattle feeding routines were traditional, and farmers selected fodder ingredients and made their own mixtures. Conventional Danish milk cattle fodder consists of a mixture of corn silage (35%), wheat-barley silage (25%), clover grass silage (20%), soybean meal or rapeseed cake (10%), and a small proportion of molasses or beet pellets, straw, vitamins and minerals. Milk from the grazing cows was transported to Tine BA (Oslo, Norway) where the fat was processed into butter.

### Diets and test fats

The test butter had different FA compositions (Table 2). The cholesterol-raising saturated fatty acids, lauric, myristic and palmitic acid, constituted 20% less of the total FAs in the G butter than the C butter (C12:0; C14:0 and C:16:0) (36.4 vs. 45.4 wt%). The G butter also had a 26% higher content of stearic acid (C18:0) (15.1 vs. 11.1 wt%), 32% higher content of *trans-*vacceninc acid (C18:1 *t*-11) (2.5 vs. 1.7 wt%), 17% higher content of oleic acid (C18:1 *c*-9) (25.8 vs. 21.5 wt%), and 33% higher content of α-linolenic acid (C18:3 n-3) (0.9 vs. 0.6 wt%) compared with C butter. Furthermore, the n-6/n-3 ratio was 45% lower in the G butter (2.9 vs. 1.6 wt%) compared with C butter. The butter was incorporated into buns. The buns were made in one batch and provided by the Department of Nutrition, Exercise and Sports in Copenhagen. During the intervention period, subjects were requested to replace part of their daily diet with three buns. The buns contained 3.5 MJ of energy, with 7.5 E% from protein, 56 E% from carbohydrates, and 36.4 E% from fat. All buns contained 13 g of butter, which yielded a fat intake from the test diet of 39 g a day (15 MJ).

**Table 2:** Fatty acid composition of the test butter from mountain-pasture grazing cows and from conventionally fed cows

	**% fatty acid of total fatty acid**	
	**Grazing**	**Conventional**
	*% wt*	
C4:0	1.0	0.8
C6:0	1.3	1.4
C8:0	1.0	1.1
C10:0	2.2	2.8
C12:0	2.6	3.4
C14:0	9.4	11.0
C14:1	0.7	1.0
C15:0	0.9	1.1
C16:0	24.4	31.0
C16:1 n-7	1.2	1.6
C20:0 (phytanic acid)	0.4	0.2
C17:0	0.6	0.5
C17:1	0.2	0.2
C18:0	15.1	11.1
C18:1 *t*-11	2.5	1.7
C18:1 n-9	25.8	21.5
C18:1 n-7	0.4	0.9
*c-*9 *t-*11 CLA	0.7	0.6
C18:2 n-6	1.5	1.8
C18:3 n-3	0.9	0.6
n-6:n-3 ration	1.6	2.9
Total SFA	58.9	64.4
Total UFA	33.9	30.0
Total MUFA	30.8	27.0
Total PUFA	3.2	3.0

### Compliance

We used the concentration of pentadecanoic acid (C15:0) in plasma as an indicator of milk fat intake, and thus of compliance, because C15:0 in serum is considered as a valid marker for intake of milk fat in humans [35-37]. In addition, dietary records and log books were used to reinforce the dietary advice and strengthen compliance, and the results of the last records were used in the calculation of dietary changes during the study. A participant was considered compliant when > 90% of the supplied buns had been eaten.

### Blood sampling and analysis

Blood samples were taken in duplicates on two consecutive days, before the intervention period and at the end of the intervention, from fasting subjects after 10 minutes of supine rest. The values from each sample set were averaged. In addition to fasting for 12 hours, participants were asked to refrain from smoking 12 hours prior to the blood sampling and to refrain from performing any extreme sport 36 hours prior to taking the sample. Furthermore, they were asked not to drink alcohol or take medicine 24 hours prior to the blood sampling. Blood for FA analysis was collected in tubes containing EDTA, which were kept on ice, and the samples were centrifuged at 4°C and 2200 × g for 15 min. All samples were stored at −80°C until they were analysed. All samples were analyzed at the Department of Human Nutrition, except the FA analyses, which were performed at the Technical University of Denmark.

### Blood lipids

We assessed serum LDL and HDL cholesterol by enzymatic colorimetric procedure (ABX Pentra LDL Direct CP and ABX Pentra HDL Direct CP respectively) on ABX Pentra 400 Chemistry Analyzer (HORIBA ABX, Montpellier, France). Total cholesterol was assessed and analyzed by enzymatic photometric procedure (CHOD-PAP from ABX Pentra Cholesterol CP) on ABX Pentra 400 Chemistry Analyzer (HORIBA ABX, Montpellier, France). The concentration of triacylglycerol (TAG) was assessed and analyzed by enzymatic colorimetric procedure (ABX Pentra Triglycerides CP) on ABX Pentra 400 Chemistry Analyzer (HORIBA ABX, Montpellier, France). The inter assay coefficients of variation (CV%) for total, LDL and HDL cholesterol, and TAG were 1.9%, 3.3%, 2.8% and 4.2%, respectively. Intra assay CV% for total, LDL and HDL cholesterol, and TAG were 0.9%, 0.9%, 1.2% and 2.6% respectively.

### Fatty acid analysis in lipids and test fats

Total lipids were extracted and analyzed from blood plasma samples as described by Tholstrup *et al.*[38] but using TAG C19:0 as an internal standard. For lipidextraction from the buns, three buns were homogenized in a kitchen blender, and three samples were randomly taken from the homogenate. The bun lipids were extracted according to the method of Folch [39], while preparation of fatty acid methyl esters (FAME) was performed as described earlier [38]. The response factor was calculated for methyl esters of phytanic acid and short-chained fatty acid, based on the response of palmitic acid methyl ester (C16:0). The butter FAME were separated on a 60-m Supelco SP-2380 column (Sigma-Aldrich AS, Brøndby, Denmark) in a HP 6890 gas Chromatograph (GC) in split mode using He as carrier gas. GC settings were: Injector temperature 260°C, split ratio 1:20, carrier flow 1.2 mL/min, detector temperature 300°C, air flow in detector 300,0 mL/min, hydrogen flow 35 mL/min. FAME were separated using a temperature program starting at 50°C and gradually increasing it to 160°C at a rate of 15°C/min; this temperature was kept for 0 min, after which the temperature was increasedto 182°C at a rate of 1°C/min. The temperature was then raised to 200°C at a rate of 10°C/min, and the oven was kept at 200°C for 15 min before the temperature was increased to 225°C. The final temperature was kept for 12 min (total runtime 61.97 min). FAME was identified with authentic standards.

### Hs-C-reactive protein

Blood for analysis of high sensitive C-reactive protein (*hs*CRP) was collected into dry tubes; samples were subsequently centrifuged at 2200 × g for 15 min at 20°C. Serum was stored at −80°C until the samples were analysed. The CRP concentrations were measured using latex immunoturbidimetry (ABX Pentra CRP CP) on ABX Pentra 400 Chemistry Analyzer (HORIBA ABX, Montpellier, France). The detection limit was 0.1 mg/L. The inter and intra assay CV% was 3.4% and 3.6%, respectively.

### Insulin, C-peptide and glucose

Blood for analysis of insulin and C-peptide was collected in dry tubes, whereas the blood samples for glucose were collected in tubes with fluoride citrate; for both serum and plasma preparation the samples were centrifuged at 2200 × g for 15 min at 20°C after coagulation. The samples were stored at −80°C until analysis. Insulin and C-peptide concentrations were measured in serum with a chemiluminescent immunometric assay on DPC Immulite 1000 (Siemens Medical Solutions Diagnostics, USA). Glucose concentrations were measured in plasma and analyzed by enzymatic colorimetric procedure (ABX Pentra Glucose HK CP) on ABX Pentra 400 Clinical Chemistry Analyzer (HORIBA ABX, Montpellier, France). The inter assay CV% for insulin, C-peptid and glucose was 5.1%, 4.3% and 1.7%, respectively. The intra assay CV% for insulin, C-peptid and glucose was 2.7%, 1.9% and 1.1%.

### Oral glucose tolerance test

A standard 75 g OGTT was administered at baseline and after 12 weeks of the study, with blood sampled for glucose and C-peptide at 0, 15, 30, 60, 90, 120 and 180 minutes [40]. Three-hour C-peptide and glucose areas under the curve (AUC) were calculated from the OGTT data, in millimol per minute per liter for glucose, and picomol per minute per liter for C-peptide. Glucose and C-peptide AUCs are the sum of the area of each time segment by C-peptide or glucose concentration. C-peptide AUC is an approximate measure of C-peptide secretion in response to a standard oral glucose load.

### Statistical analysis

For both diet treatments, baseline characteristics were summarized by means of averages, standard deviations and minimum/maximum values. Pearson correlation coefficients with corresponding p values for testing whether or not the correlation is 0 were calculated between baseline values. The outcome variables total cholesterol, LDL, HDL, TAG, hsCRP, insulin, C-peptide, glucose and phytanic acid were analyzed using an analysis of covariance (ANCOVA) model including the treatments (the explanatory variable of interest) and adjusting for differences between groups by including the baseline level as well as the relevant covariates age, gender, smoker and weight, which may be possible confounders when evaluating blood parameters. The treatment effect was evaluated by means of an F-test. Treatment differences were reported in terms of estimated mean levels with corresponding standard errors.

Analysis of the OGTT data was carried out using a linear mixed ANCOVA including the interaction between time and diet treatment in order to assess if time modified the effect of the diets during the three-hour period of OGTT. Period effects as well as covariates adjusting for group differences in age and BMI were included in the model. Random effects were included to capture between-subject variation. The time course of the estimated treatment effects for both diets was shown graphically with corresponding standard errors. An approximate F-test was used to evaluate the diet-time interaction and, if non-significant, another approximate F-test was used to evaluate if there was a time-independent diet effect. In addition, diet effects in glucose AUC and C-peptide AUC were assessed using ANCOVA, adjusting for the baseline AUC.

For all models validation was based on graphical assessment of residual plots and normal probability plots. In the event departures were detected, the outcome variables in question were transformed using the logarithm transformation.

P values were evaluated at a 5% significant level. The analyses were carried out using PROC MIXED procedure in Statistical Analysis System (SAS), version 9.1 (SAS Institute, Cary, NC).

## Results

### Compliance

There was no difference in plasma C15:0 after the intervention, and the average increase in C15:0 from baseline was around 20% in both groups, which indicates the consumption of a similar amount of dairy products in both groups during the intervention (data not shown). Counting the number of buns eaten showed a mean compliance of 99.18 ± 1.98 % in all groups together and of 99.37 ± 1.75 % and 98.96 ± 2.23 % in the G and C group, respectively. The number of buns not eaten did not differ between the groups (p=0.54).

### Dietary intake and body weight

Both groups had a minor weight gain during the intervention, (0.89 ± 1.39 and 0.93 ± 0.78 kg for G and C, respectively), but the weight gain did not differ between the groups when expressed either as kg body weight (p=0.80) or BMI (p=0.55). A dietician evaluated all dietary records. One record from the G group was incomplete and therefore excluded from the statistical analysis. The energy and macronutrient content in the participants’ daily diet during the intervention were calculated from the dietary records (Table 3). The distribution (% energy) of protein (p=0.89), carbohydrates (p=0.69), and total fat (p=0.40) did not differ between the two groups.

**Table 3:** **Macronutrient intake after 6 weeks of the intervention diet**^**1**^

**% of energy**	**Grazing (*n*=19)**	**Conventional (*n*=18)**
Protein	14.3 ± 0.4	14.3 ± 0.4
Carbohydrates	47.5 ± 1.3	46.7 ± 1.3
Total Fat	34.0 ± 0.9	35.1 ± 0.9
Saturated FAs	16.0 ± 0.7	15.8 ± 0.7
Monounsaturated FAs	10.2 ± 0.4	10.6 ± 0.4
Polyunsaturated FAs	4.0 ± 0.2	3.6 ± 0.2

^1^ All values are estimated means ± SE. *FAs* Fatty acids, *Grazing* milk from grazing cows, *Conventional* milk from conventionally fed cows. Values were obtained from 3-day weighed food records. One dietary record was incomplete and therefore excluded from the statistical analysis. No significant differences between the two groups were found (ANCOVA).

### Blood samples

We found strong correlations between plasma phytanic acid at baseline and both total (r^2^=0.42, p<0.0001) and LDL cholesterol (r^2^=0.35, p=0.0001). The correlation between the known marker of dairy-fat intake, plasma C15:0 and both total cholesterol and LDL cholesterol was weaker (r^2^=0.14, p=0.02 and r^2^=0.11, p=0.04, respectively), although there was a strong correlation between plasma C15:0 and phytanic acid (r^2^=0.44, p<0.0001) (Table 4).

**Table 4:** Pearson’s correlations coefficients between baseline variables

	**Phyt**	**HDL**	**LDL**	**Total CH**	**Glucose**	**Insulin**	**hsCRP**	**TAG**	**C15:0**
Phyt	1.0	0.24	0.6^1^	0.65^1^	0.16	0.01	0.28	0.29	0.67^1^
HDL		1.0	0.25	0.54^4^	−0.36^4^	−0.45^4^	−0.27	−0.46^4^	0.11
LDL			1.0	0.93^4^	−0.14	−0.11	0.16	0.21	0.34^2^
Total CH				1.0	−0.16	−0.2	0.07	0.15	0.38^3^
Glucose					1.0	0.40^4^	0.49^4^	0.59^1^	0.41^4^
Insulin						1.0	0.12	0.46^4^	−0.03
hsCRP							1.0	0.46^4^	0.36^4^
TAG								1.0	0.33^4^
C15:0									1.0

*hsCRP* high sensitive C-reactive protein, *TAG* Triacylglycerol, *CH* cholesterol, *Phyt* Phytanic acid.

C15:0, Plasma pentadecanoic acid. n = 38. ^1^ Significantly correlated *P≤0.0001*. ^2^ Significantly correlated *P=0.04.*

^3^ Significantly correlated *P=0.02*. ^4^ Significantly correlated *P<0.05*.

**Figure 1:**
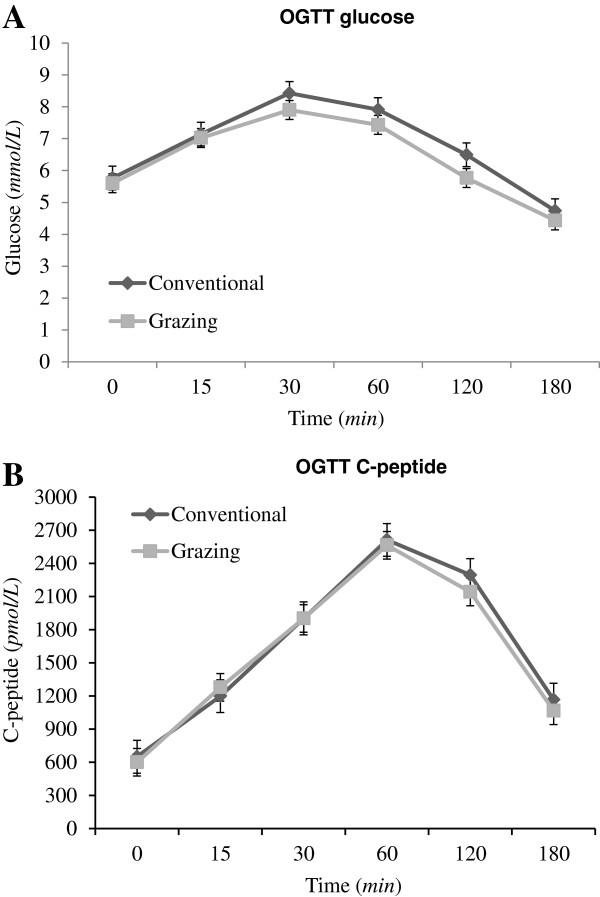
**Oral glucose tolerance test (OGTT) measurements performed after 12 weeks of intervention.** All values are means ± SE. *Grazing* milk from grazing cows (*n*=20). *Conventional* milk from conventionally fed cows (*n*=18). Glucose tolerance was measured using a standard 75 g oral glucose-tolerance test. There was no significant difference between areas under the curve (AUC). There was no significant difference in the time course of glucose and C-peptide concentration after the two different test fats or in the mean glucose and C-peptide concentration according to the repeated-measures analysis of variance.

TAG, *hs*CRP, insulin, glucose, C-peptide AUC, and glucose AUC required logarithm transformation. No significant differences were observed between G and C diet in serum total cholesterol (p=0.38), LDL cholesterol (p=0.55), HDL cholesterol (p=0.51), TAG (p=0.36), hsCRP (p=0.54), insulin (p=0.60), C-peptide (p=0.91), plasma glucose (p=0.63) or plasma phytanic acid (p=0.15) (Table 5). Analysis of the OGTT data revealed neither a significant treatment-time interaction nor a significant treatment effect for glucose (p=0.52 and p=0.27, respectively) and for C-peptid (p=0.43 and p=0.98, respectively) (Figure 1). There was no significant difference between the AUCs for either glucose (p=0.89) or C-peptide (p=0.65) after the test diets (Figure 1).

**Table 5:** Effects of the 12-week dietary intervention

	**Grazing (*n*=20)**	**Conventional (*n*=18)**
Total cholesterol, (*mmol/L*)	5.9 ± 0.10	5.8 ± 0.11
LDL cholesterol, *(mmol/L)*	3.4 ± 0.09	3.3 ± 0.09
HDL cholesterol, *(mmol/L)*	1.5 ± 0.04	1.4 ± 0.04
Triacylglycerol, *(mmol/L)*	1.2 ± 1.08	1.1 ± 1.09
hsCRP, (*mg/L)*	0.8 ± 1.36	0.6 ± 1.41
Insulin, *(pmol/L)*	32.0 ± 1.11	30.0 ± 1.12
C-peptide, *(pmol/L)*	586.1 ± 1.05	582.1 ± 1.05
Glucose, *(mmol/L)*	5.6 ± 1.01	5.7 ± 1.02
Phytanic acid, *(μM)*	3.6 ± 0.17	3.3 ± 0.18

All values are means ± SE. *hsCRP* high sensitive C-reactive protein, *Grazing* milk from grazing cows, *Conventional* milk from conventionally fed cows. No significant differences between the two diet groups were found (ANCOVA).

There were no significant differences between groups in the erythrocyte FA composition (data not shown).

## Discussion

This study was designed to compare the effects of butter produced from milk from mountain-pasture grazing cows with conventional Danish butter on risk markers of CVD and type-2 diabetes. There were no differences between diets with regard to effect on the metabolic parameters or on *hs*CRP.

Despite the fact that the cholesterol-raising SFAs were decreased with 20% (45.4 to 36.4 % of total FAs) in the G diet, this did not lead to lower plasma total and LDL cholesterol concentrations. This is in agreement with results from a study by Tholstrup *et al.* where the cholesterol-raising SFAs, especially palmitic acid, was replaced with mainly oleic and stearic acid [41]. In contrast, Noakes *et al.* found that consumption of modified dairy products produced through the substitution of cholesterol-raising SFAs with oleic and linoleic acid, significantly reduced total- and LDL cholesterol with 4.3% and 5.3%, respectively [42]. This study reported a substantially higher change in PUFA (8 g/100 g FA), which is known to lower plasma LDL cholesterol more than do MUFA [43,44], and may be the main reason for the LDL cholesterol lowering effect [42]. This is supported by another study, which reported a decrease plasma total-and LDL cholesterol by consuming a similar modified butter with a high increase in PUFA (6.5 g/100 g FA) [45]. In addition, a combination of a relatively lower SFA and substantially higher linoleic and oleic acid in Noakes *et al.*[42] compared with our study could explain why the hypocholesterolemic effects of oleic and linoleic acid were successfully “expressed”.

A minor increase in ruminant *trans* FA (rTFA) was a side effect of manipulating the cattle fodder. A study by Tholstrup *et al.*[46] observed that the consumption of rTFA-enriched butter (3.6 g/d) lowered total and HDL cholesterol by 6% and 9% respectively, compared with butter low in rTFA and high in SFA. However, the authors concluded that these effects may have been partly attributable to the higher MUFA and lower SFA content in the rTFA-enriched butter, rather than to the effects of rTFA alone. Two recent clinical studies found an increase in plasma LDL cholesterol in conjunction with high daily rTFA intakes: 10–12 g/d, representing 3.6-5% of energy [47,48]. However, an intake of 10–12 g/d rTFA/d is very high in practical terms. The concentration of rTFA in dairy products exists in quantities that are too small to have any effect. Thus, it is unlikely that the slight difference in *trans* FA in this study may have affected the results.

The intake of fresh grass also slightly elevated the concentration of CLA in the G diet, as expected from earlier studies [17,18]. CLA has been reported to reduce aortic atherosclerosis in animal studies [49,50]. However, human study failed to find any effect of milk CLA on cholesterol. One study in which the CLA content was enriched by rumen technology to provide 1.42 g/d of c-9, t-11 CLA in dairy products failed to affect LDL cholesterol level [27], which is in line with results from another study with a daily intake of 4.22 g milk CLA [23]. In addition, it is unlikely that the slight difference in CLA and the lower concentration of CLA in the current study may have affected the results.

The markedly higher intake of chlorophyll-containing green fodder in grazing cows also resulted in a high phytanic acid content in the milk. In a previous study conducted by our group [51], we found a significant increase in plasma phytanic acid in both groups, verifying that intake of dairy products with relatively low concentrations of phytanic acid increase circulating phytanic acid concentration. This finding seems to be in agreement with observations made in this study. In the present study we found surprisingly strong correlations between phytanic acid at baseline, total cholesterol (r^2^=0.42, p<0.0001) and LDL cholesterol (r^2^=0.35, p=0.0001). It seems likely that the baseline phytanic acid concentration reflects habitual dairy fat intake, which means that the strong correlations could be due to the hypercholesterolemic effect of dairy fat. This interpretation is supported by the fact that plasma phytanic acid and plasma C15:0 correspond very strongly at baseline (r^2^=0.44, p<0.0001). However, the correlations between plasma C15:0 and both total cholesterol (r^2^=0.14, p=0.02) and LDL cholesterol (r^2^=0.11, p=0.04) were substantially weaker than those observed for phytanic acid. This suggests that phytanic acid increases LDL cholesterol beyond levels that can be explained solely from dairy fat intake, indicating that phytanic acid may have specific LDL cholesterol rising effects.

We observed no differences in effects on glucose or insulin concentration between the groups. Prior to the present study, the available literature supported the hypothesis that phytanic acid might have preventive effects on metabolic dysfunctions related to the development of the metabolic syndrome [30-33]. In particular the RXR-activating ability of phytanic acid is of interest, since RXR is activated by concentrations at the same level as the phytanic acid concentrations reached in plasma in this study (21). It has been shown that drugs that activate RXR and PPAR-α have a marked impact on whole body metabolism and act as insulin-sensitizers or as hyperlipidemic agents [52,53], while it is not clear to what extent dietary changes in natural agonist concentrations are of physiological importance in humans.

In the current study, milk fat from mountain-pasture grazing cows which represents summer whole milk with a SFA:MUFA:PUFA proportion of 59:31:3 was more similar to conventional milk FA composition of 64:27:3 than predicted. In addition, the FA composition of conventional milk FA in the current study was more consistent with consumer perceptions of a healthy diet and with public health recommendations compared to conventional milk from 1998 with the composition of 75:22:4 [41]. This could be due to a higher amount of grass-based products in the cows’ feed, and could explain why no effect is observed in the current study.

The strengths of our study include its controlled and randomized design, the long intervention period, follow-up of dietary compliance during the study, and the use of a validated dietary marker to monitor the dairy-fat intake. The study was also designed to investigate dietary changes caused by an increased intake of dairy products in a realistic setting, and the plasma phytanic acid concentration in this study population occurred in a concentration which has physiological relevance as a modulator of RXR-activity. Among the limitations were the relatively high intakes of butter during the intervention. Also breed of the cows may have played a role; however there is no evidence that FA composition is differently influenced by breed *per se.* The health effect of milk from pasture grazing cows should be further investigated. Furthermore, it could be interesting to elucidate the association between phytanic acid and the PPAR-α activity to better prove the effects of phytanic acid on plasma cholesterol.

## Conclusion

Lack of effects on blood lipids and inflammation indicates that dairy products from mountain-pasture grazing cows are not healthier than products from high-input conventional systems. Considering the strong correlation between LDL cholesterol and phytanic acid at baseline, it may be suggested that phytanic acid increases total and LDL cholesterol. Additional studies are necessary to clarify the effect of phytanic acid on risk markers of the metabolic syndrome.

## Abbreviations

FA: Fatty acid; SFA: Long-chain saturated fatty acid; MUFA: Monounsaturated fatty acid; PUFA: Polyunsaturated fatty acid; CLA: Conjugated linoleic acid; RXR: Retinoid X receptor; PPAR-α: Peroxisome proliferatoractivated receptor-*α*; CVD: Cardiovascular diseases; OGTT: Oral glucose tolerance test; C15:0: Pentadecanoic acid; TAG: Triacylglycerol; CV: Coefficient of variation; hsCRP: High sensitive C-reactive protein; AUC: Area under the curve; SAS: Statistical Analysis System; rTFA: ruminant trans fatty acid.

## Competing interests

The authors declare that they have no competing interests.

## Authors’ contributions

LBW: Conducted research, performed statistical analyses and drafted the original paper. LIH: Developed the initial ideas, analyzed and interpretated data and edited paper. MR: Conducted research. SKJ: Provided milk for the project. RAP: Carried out the practical work with the human study. TD: Analyzed data. TT: Developed the initial ideas, designed the study and was study project leader, interpreted results and participated in the writing of the paper. All authors have read and approved the final manuscript.
